# Song recognition in Floridian *Ormia ochracea* reflects multidimensional temporal tuning

**DOI:** 10.1242/jeb.252806

**Published:** 2026-09-15

**Authors:** Lauren J. Bitner, Jimena A. Dominguez, Luke Bemish, Quang Vu, Julia F. Morgan, David A. Gray, Andrew C. Mason, Norman Lee

**Affiliations:** ^1^ Department of Biology, St. Olaf College, Northfield, MN 55057, USA; ^2^ Department of Biology, California State University Northridge, Northridge, CA 91330, USA; ^3^ Department of Biological Sciences, University of Toronto Scarborough, Scarborough, ON, Canada, M1C 1A4

**Keywords:** Temporal pattern recognition, Auditory processing, Eavesdropper, Acoustic communication, Phonotaxis

## Abstract

Acoustic communication signals often contain a complex temporal structure, yet how this temporal structure contributes to signal recognition is poorly understood, particularly in eavesdropping receivers. The parasitoid fly *Ormia ochracea* localises host crickets by eavesdropping on their calling songs. In Florida, preferred host songs consist of sound pulses repeated at ∼50 pulses s^−1^, and flies exhibit matching preferences. However, it is unclear whether this preference reflects sensitivity to individual temporal parameters or to derived temporal relationships that emerge from their combination. We independently varied pulse duration and interpulse interval across a broad stimulus space and quantified tethered-walking phonotaxis using a switch-following paradigm. Behavioural responses formed a structured tuning surface, with elevated responses for pulse durations of ∼5–15 ms across a broad range of interpulse intervals, together with high performance along a diagonal corresponding to 50 pulses s^−1^. Responses failed to collapse across stimuli sharing the same pulse rate or pulse period, indicating that these parameters alone do not determine recognition. Models allowing behavioural responses to vary jointly across combinations of temporal parameters substantially outperformed models based on single parameters. Although pulse duration strongly constrained recognition, behavioural responses also varied systematically with interpulse interval and could not be explained by pulse duration, interpulse interval, pulse rate, pulse period or duty cycle alone. The apparent preference of *O. ochracea* for ∼50 pulses s^−1^ therefore represents one component of a broader temporal recognition landscape rather than recognition based on pulse rate alone.

## Teaser

**Summary:** The acoustic parasitoid fly *Ormia ochracea* is behaviourally tuned to combinations of durations and interpulse intervals of cricket songs in the context of host recognition.

## INTRODUCTION

Acoustic communication plays a central role in the behaviour of many animals, allowing for the transmission of information from senders to receivers (Bradbury and Vehrencamp, 1998). These signals can encode relatively static information such as species identity, sex or individuality, as well as more dynamic information related to motivation, condition or behavioural state (Gerhardt and Huber, 2002). Acoustic signals facilitate a wide range of behavioural interactions, including the coordination of reproductive behaviours (Andersson, 1994; Searcy and Nowicki, 2005), aggressive encounters and territorial defence (Searcy and Nowicki, 2005), predator avoidance (ter Hofstede and Ratcliffe, 2016), and prey or host localisation (Walker, 1993). For communication to function effectively in these contexts, receivers of communication signals must detect, discriminate and recognise behaviourally relevant signals within complex natural environments (Feng and Ratnam, 2000; Miller and Bee, 2012). While these recognition processes are typically studied in the context of intended receivers (Baker et al., 2019), communication signals may also be intercepted by unintended eavesdroppers such as predators and parasitoids that exploit these signals to locate their prey or host (Bernal and Page, 2023; Zuk and Kolluru, 1998). How then do these predators and parasitoids recognise the same communication signals?

The early stages of auditory processing involve the frequency analysis of auditory input (Hedwig, 2016; Mason and Faure, 2004). However, while spectral tuning determines which signals are relevant, recognition in many systems depends critically on the extraction of the temporal structure of communication signals. Multiple conceptual frameworks have been proposed to explain temporal pattern recognition, which differ in both the computations they perform and their underlying neural implementations. One class of models emphasises feature detection, in which neurons are selectively tuned to specific temporal attributes of communication signals, such as pulse duration (PD) (Leary et al., 2008), interpulse interval (IPI) (Deutsch et al., 2019; Edwards et al., 2008; Ronacher and Stumpner, 1988) or pulse rate (Schildberger, 1984; Thorson et al., 1982). Such selectivity can arise through a temporal integration process where neuronal responses accumulate over successive sound pulses, producing sensitivity to pulse rate or to a threshold number of sound pulses occurring at the appropriate interval (Alder and Rose, 1998; Rose et al., 2011). More formally, this class of computations can be described using spectrotemporal filters (Hennig, 2009), such as Gabor-like filters, which are jointly tuned to duration and temporal modulation rate and can drive temporal selectivity (Hennig et al., 2014; Ronacher et al., 2015). Recent work (Mann et al., 2025) has demonstrated that such filter-like selectivity can emerge from biologically realistic circuits through the interaction of excitation and inhibition, providing a unifying framework that links feature tuning to underlying neural dynamics. A second class of models emphasises cross-correlation or template-matching computations, in which the temporal features of signals are compared with an internal template over a defined time window (Hennig, 2003; Kostarakos and Hedwig, 2015). These models operate at the level of global pattern matching and assess the similarity between auditory input and expected temporal structure. A third class of mechanisms is based on temporal selectivity that arises from delay-line and coincidence detection mechanisms, in which inputs are temporally offset and subsequently recombined such that neurons respond maximally when signals arrive simultaneously (Jeffress, 1948; Schoneich et al., 2015). This framework provides an explicit mechanism for detecting specific IPIs or pulse periods through circuit-level timing computations. While these conceptual frameworks differ in their level of description, they share the common goal of extracting temporal regularity from signals. Importantly, accumulating computational and neurophysiological evidence suggests that temporal selectivity may emerge from sequential processing involving excitatory and inhibitory interactions within local neural circuits (Clemens et al., 2021; Schoneich et al., 2015).

Despite substantial progress in understanding the neural mechanisms underlying signal recognition in intended receivers (Baker et al., 2019; Hennig et al., 2014; Kostarakos and Hedwig, 2015; Ronacher et al., 2015; Schöneich, 2020), it is unclear whether these same mechanisms operate in unintended receivers such as predators and parasitoids that exploit communication signals. In these systems, recognition serves fundamentally different functions (locating prey or hosts versus facilitating communication between conspecifics), which may impose distinct selective pressures on sensory processing (Wikle et al., 2025). Consequently, it is unknown whether eavesdropping receivers rely on similar feature-based, interval-based or periodicity-based mechanisms, or whether alternative strategies have evolved to extract behaviourally relevant information from communication signals.

The acoustic parasitoid fly *Ormia ochracea* is a striking example of an eavesdropper that relies on the communication signals of field crickets. Male crickets produce calling songs to attract potential mates (Alexander, 1957; Gerhardt and Huber, 2002). At the same time, gravid female flies rely on these species-specific calling songs to locate host crickets (Cade, 1975; Zuk and Kolluru, 1998) for the development of their larval young (Adamo et al., 1995; Dominguez et al., 2025). The auditory system of female *O. ochracea* is tuned to the ∼5 kHz carrier frequency of host cricket calling songs, enabling detection of these songs over distances exceeding 50 m (Robert et al., 1992; Wikle et al., 2025). Following detection, flies exhibit remarkable sound localisation abilities and can orient to sound sources with an accuracy of 2 deg azimuth (Mason et al., 2001). Once oriented toward a potential host, flies engage in both flying (Müller and Robert, 2001) and walking phonotaxis (Lee et al., 2009; Mason et al., 2005) to the source location. Beyond sound detection and localisation, behavioural studies demonstrate that flies can maintain preferences for host song temporal patterns across a range of ambient temperatures, consistent with physiological temperature coupling between senders and receivers (Jirik et al., 2023), although expression of this potential may be limited under some field conditions (Rossi et al., 2024). While these findings indicate that flies respond to temporal variation in host songs, the temporal parameters governing recognition are unclear.

In Florida, *O. ochracea* are locally adapted to recognise and prefer the calling songs of the field cricket *Gryllus rubens* (Gray et al., 2007; Walker, 1993). These crickets produce calling songs that consist of long trills with ∼10 ms PDs and ∼10 ms IPIs, resulting in a pulse rate of ∼50 pulses s^−1^ at an ambient temperature of 21°C (Walker, 1962; Izzo and Gray, 2004). Past experiments indicate that Floridian *O. ochracea* also exhibit preferences for a pulse rate of 50 pulses s^−1^ (Jirik et al., 2023; Lee et al., 2019). However, it is unclear whether this preference arises from sensitivity to individual temporal parameters, such as PD or IPI, or from sensitivity to derived temporal relationships, such as pulse rate, pulse period or duty cycle that emerge from the combination of these parameters. Here, we asked whether song recognition can be reduced to a single scalar description of temporal pattern, or whether behavioural responses instead exhibit multidimensional temporal structure.

**Fig. 1.**
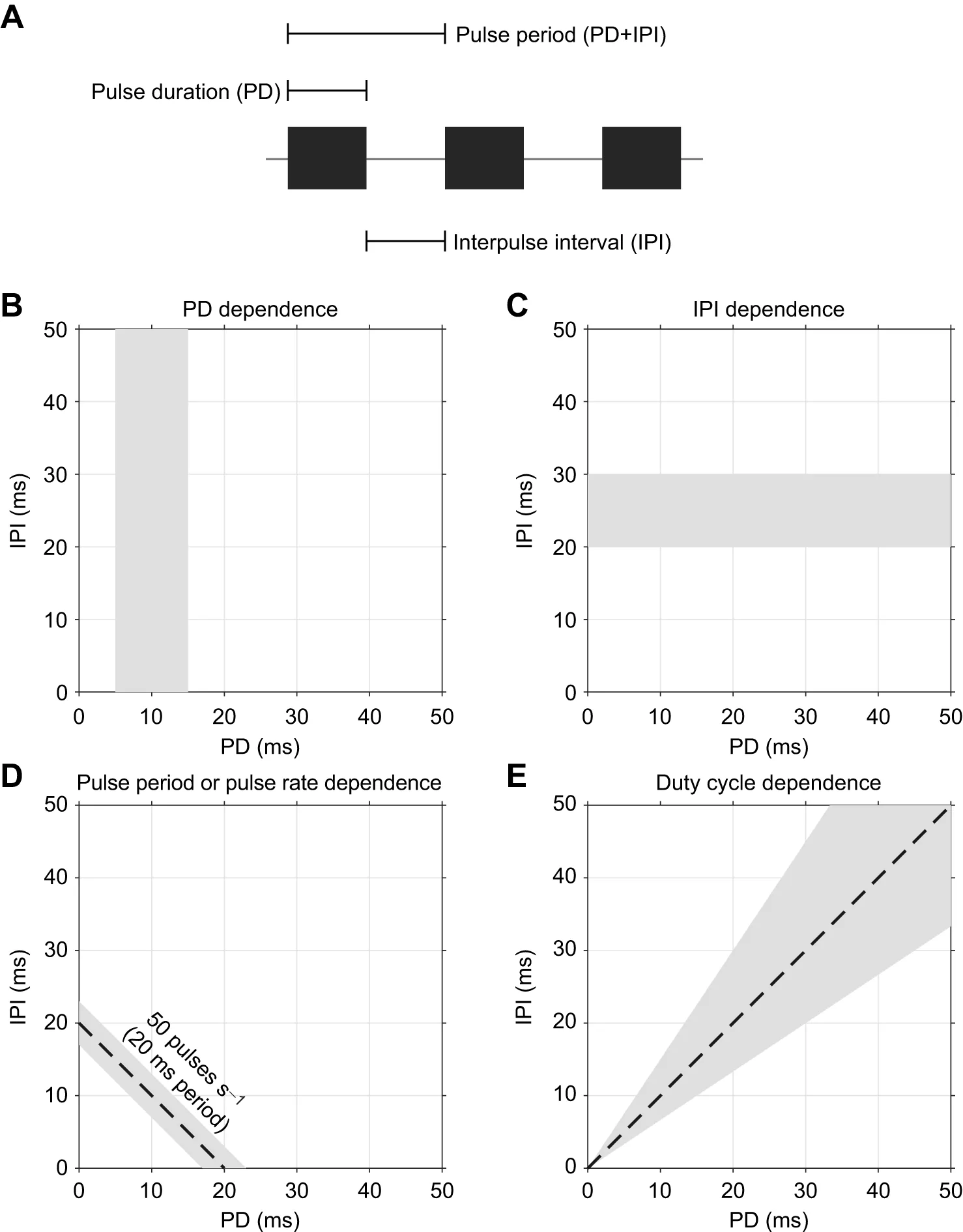
**Predicted behavioural response patterns under alternative hypotheses of temporal pattern song recognition in *Ormia ochracea*.** (A) Schematic illustration of the temporal parameters used to describe acoustic stimuli modelled after host cricket songs. Pulse duration (PD) is the duration of each sound pulse, interpulse interval (IPI) is the silent interval between successive pulses, and pulse period is the interval between pulse onsets (PD+IPI). Duty cycle is the proportion of each pulse period occupied by sound [PD/(PD+IPI)], and pulse rate is the reciprocal of pulse period. (B–E) Schematic predictions for behavioural responses plotted in the PD–IPI stimulus space if song recognition depends primarily on different temporal parameters. (B) Dependence on PD predicts a vertical region of high performance (grey shading), such that stimuli sharing preferred PDs elicit similar responses across IPIs. (C) Dependence on IPI predicts a horizontal region of high response across PDs. (D) Dependence on pulse period, and equivalently pulse rate, predicts a negatively sloped diagonal because combinations of PD and IPI with the same sum have the same pulse period; the illustrated 20 ms period corresponds to 50 pulse s^−1^. (E) Dependence on duty cycle predicts a positively sloped diagonal because PD and IPI must covary proportionally to maintain a constant duty cycle; the illustrated relationship corresponds to a 50% duty cycle. Predicted response patterns are schematic and are intended to illustrate the geometry expected under each hypothesis rather than specific empirically determined tuning ranges.

These alternative descriptions make distinct predictions for the structure of behavioural responses across PD–IPI stimulus space (Fig. 1). If responses depend primarily on PD, high switch-following performance should occur within a restricted range of PDs across a broad range of IPIs, producing a vertical band in PD–IPI space. Conversely, predominant sensitivity to IPI should produce a horizontal band. Pulse period is defined as the interval from the onset of one pulse to the onset of the next (PD+IPI), while pulse rate is its reciprocal [1/(PD+IPI)]. Thus, pulse period and pulse rate are mathematically equivalent descriptions of the same temporal relationship, expressed in different units. If responses depend primarily on pulse period, or equivalently pulse rate, high performance should follow a negative diagonal corresponding to PD–IPI combinations with the same period. Finally, sensitivity to duty cycle should produce a positive diagonal, because a constant ratio of sound duration to total period requires PD and IPI to covary proportionally. A response surface that does not conform to any single one of these predictions would instead indicate that behavioural recognition cannot be reduced to a single temporal parameter. We tested these alternatives by independently varying PD and IPI across a broad stimulus space and asked whether behavioural responses collapse onto one of these predicted patterns or instead exhibit a multidimensional structure. More broadly, determining how eavesdropping receivers respond to variation in the temporal structure of communication signals can provide insight into the principles underlying signal recognition and how these may differ between eavesdroppers and intended receivers.

## MATERIALS AND METHODS

### Animals

Walking phonotaxis experiments were conducted on gravid female *Ormia ochracea* (Bigot 1889) from a laboratory colony originally derived from Gainesville, Florida, USA. The colony was maintained through manual parasitisation of *Acheta domesticus* crickets following previously described methods (Dominguez et al., 2025). Flies were reared in temperature-, humidity- and light-controlled environmental chambers (model DROS52503, Power Scientific Inc., Pipersville, PA, USA) set to a 12 h light:12 h dark cycle at 75% humidity, and provided with butterfly nectar (The Birding Company, Yarmouth, MA, USA) *ad libitum*. A total of 52 gravid female flies were included in this study.

### Behavioural measurements

#### Experimental setup

Walking phonotaxis responses were recorded using a spherical treadmill system (Lott et al., 2007). The treadmill was positioned 25 cm away from two Avisoft Bioacoustic ultrasonic speakers (Vifa Speaker, part #60108, Glienicke/Nordbahn, Germany) positioned at ±45 deg azimuth to the left and right of the treadmill system relative to the forward orientation of the fly (0 deg azimuth). Sound presentation and treadmill data acquisition were synchronised using a National Instruments data acquisition system (NI USB-6363, Austin, TX, USA) interfaced with a custom MATLAB (R2018a, The MathWorks Inc.) graphical user interface (StimProg V6; https://github.com/Ormia/Stimprog).

Locomotor data captured as *x* and *y* pixel values were converted to centimetres for subsequent data analysis (see below). The experimental setup was housed in an acoustically dampened sound box within a dark room kept at 30% humidity and 21°C.

#### Acoustic stimuli

Synthetic acoustic stimuli were generated in MATLAB using a custom script (https://github.com/Ormia/Stimprog) and then converted to analog signals using the National Instruments data acquisition hardware (NI USB-6251, 44,100 Hz), amplified (SLA1 2-Channel 140W Amplifier, ART, Niagara Falls, NY, USA), and broadcast through two Avisoft Bioacoustic ultrasonic speakers (Vifa, part #60108). Sound levels were controlled using programmable attenuators (Tucker Davis Technologies System 3 PA5, Alachua, FL, USA). All stimuli were calibrated at the location of the fly using a probe microphone (B&K Type 4182, Nærum, Denmark) connected to a sound level meter (B&K Type 2250). Each speaker was calibrated to 75 dB SPL (re. 20 μPa). Each song consisted of a train of sound pulses; individual pulses were composed of a 5 kHz sinusoidal carrier gated by 1 ms on/off cosine-squared ramps. Pulse trains were defined by two temporal parameters: PD, corresponding to the total duration of each sound pulse (including the ramps), and IPI, the silent interval between successive pulses. PD and IPI were independently varied across a set of values (2, 3, 5, 7, 10, 13, 15, 20, 25, 30, 35, 40, 45 and 50 ms), resulting in a total of 196 acoustic stimuli spanning a broad temporal parameter space. Sampling density varied across conditions (range: 35 to 1245 trials per stimulus), and this variation was accounted for using inverse-frequency weighting during model fitting (see below).

#### General experimental protocol

Flies were anaesthetised on ice for 5 min prior to experimentation and then tethered using low melting point wax following established methods (Jirik et al., 2023; Lee et al., 2019). Flies were then mounted onto the spherical trackball system and allowed to acclimate to the behavioural testing conditions for a period of 10 min before testing. Experiments were conducted in a dark room under IR illumination, and behaviour was monitored using a camera equipped with a macro lens (Nikon AF Micro NIKKOR 105 mm f/2.8 D, Tokyo, Japan).

Test stimuli were presented for a total of 5 s: 2.5 s from one speaker followed by a switch to the opposite speaker for 2.5 s (Jirik et al., 2023; Lee et al., 2019). For each individual, the direction of switching (left-to-right or right-to-left) was fixed but randomly assigned. Each experiment began with four presentations of a reference stimulus (two left-to-right and two right-to-left). The reference stimulus consisted of a synthetic song with a 10 ms PD and 10 ms IPI, modelled after the calling song of the preferred host cricket, *G. rubens*, for Floridian *O. ochracea*. A subject was considered responsive if the total walking distance exceeded 1 cm. Only flies that responded to all initial reference stimulus presentations were included in subsequent testing. In total, 52 flies contributed 9413 behavioural trials within the prespecified PD–IPI stimulus grid.

### Data analysis

The switch-following index (SFI) quantified how reliably flies tracked the active sound source before and after the switch in stimulus broadcast location. Locomotor trajectories were smoothed using a moving-average filter, and instantaneous angular heading was calculated from frame-to-frame changes in *x*–*y* position:

(1) $$\theta_{\mathrm{heading}}(t)=\mathrm{atan}2(dy,dx).$$

At each time point, heading alignment relative to the active speaker was calculated as:

(2) $$\mathrm{Alignment}(t)=\cos(\theta_{\mathrm{heading}}-\theta_{\mathrm{target}}),$$

where θ_target_ corresponded to the angular location of the active speaker (−45 deg or +45 deg, depending on the stimulus period and switch direction). Time points at which walking velocity was below 0.2 cm s^−1^ were excluded. Mean alignment was calculated separately for the pre-switch and post-switch periods when at least one valid heading measurement remained within the corresponding period.

SFI was calculated as:

(3) $$\mathrm{SFI}=\frac{{\bar{\mathrm{Alignment}}}_{\mathrm{pre}}+{\bar{\mathrm{Alignment}}}_{\mathrm{post}}}{2},$$

where ${\bar{\mathrm{Alignment}}}_{\mathrm{pre}}$ and ${\bar{\mathrm{Alignment}}}_{\mathrm{post}}$ are the mean alignment values during the pre-switch and post-switch periods, respectively. Higher SFI values indicate stronger tracking of the active speaker across the speaker transition. SFI was calculated only for trials in which alignment could be calculated during both periods; we refer to these as complete-period trials.

Because failure to obtain an alignment value may itself reflect a stimulus-dependent failure to sustain measurable phonotaxis, we analysed response availability separately. A trial was classified as available for SFI analysis when alignment could be calculated during both the pre-switch and post-switch periods. Response availability was modelled across all 9413 trials using a binomial generalised additive mixed model with a tensor-product smooth of PD and IPI, standardised trial order and speaker-switch direction as covariates, and fly identity as a random-effect smooth (Fig. S1). Inverse-frequency PD–IPI cell weights were calculated across all 9413 trials using the same weighting procedure as in the SFI analysis and rescaled to have a mean of one. The model was fitted using restricted maximum likelihood.

To test alternative hypotheses about song temporal pattern recognition, we modelled SFI using generalised additive mixed models (GAMMs; mgcv package in R). The primary analysis comprised the 6454 trials in which alignment could be calculated during both the pre-switch and post-switch periods. Individual trial was the unit of analysis. Trial order was defined as the sequential stimulus-presentation number within each fly's experiment. Trial order and the continuous temporal predictors were mean centred and divided by their standard deviation before model fitting. Standardisation parameters were calculated from all 9413 trials within the PD–IPI stimulus grid before selecting the trials included in the SFI analysis. All candidate models included standardised trial order, speaker-switch direction (LR or RL) and a random-effect smooth for fly identity [s(fly_id, bs=“re”)] to account for repeated measurements within flies.

Sampling density varied across the PD–IPI stimulus grid because individual flies may have only experienced subsets of the 196 stimulus combinations (due to dropouts) and the PD=10 ms, IPI=10 ms reference stimulus was presented more frequently. We therefore assigned each trial an inverse-frequency weight based on its PD–IPI cell and rescaled the weights to have a mean of one. This gave each stimulus combination equal total weight. Identical trials, weights and common model terms were used for every candidate model.

Candidate models represented alternative hypotheses about the temporal structure associated with behavioural recognition rather than a nested sequence of increasing complexity (Table 1). One-dimensional models tested dependence on PD, IPI, pulse period or duty cycle. Additive models tested whether pairs of temporal parameters contributed independently. Tensor-product smooths estimated joint two-dimensional response surfaces in which the relationship between one temporal parameter and SFI could vary with the other. One-dimensional smooths were specified using s() with a basis dimension of *k*=10. Joint response surfaces were specified using te() with marginal basis dimensions of *k*=*c*(8, 8). The selected model showed no evidence that these basis dimensions were inadequate (*k*-index=1.015, *P*=0.858).

**Table 1.** Comparison of candidate generalised additive mixed models (GAMMs) representing alternative descriptions of temporal pattern recognition in Florida *Ormia ochracea*

Rank	Hypothesis	Temporal predictor term(s)	d.f.	AIC	ΔAIC	Akaike weight
1	Joint PD–IPI response surface	te(PD_*c*, IPI_*c*, *k*=*c*(8, 8))	97.9	3867.4	0.0	0.753
2	Joint duty cycle period response surface	te(duty_*c*, period_*c*, *k*=*c*(8, 8))	91.8	3869.7	2.2	0.247
3	Additive pulse period and PD effects	s(period_*c*, *k*=10)+s(PD_*c*, *k*=10)	69.0	4110.0	242.6	<0.001
4	Additive duty cycle and pulse period effects	s(duty_*c*, *k*=10)+s(period_*c*, *k*=10)	69.5	4194.1	326.6	<0.001
5	Additive PD and IPI effects	s(PD_*c*, *k*=10)+s(IPI_*c*, *k*=10)	67.3	4288.3	420.8	<0.001
6	PD dependence	s(PD_*c*, *k*=10)	60.6	4655.8	788.4	<0.001
7	Pulse period dependence	s(period_*c*, *k*=10)	60.8	5405.7	1538.3	<0.001
8	Duty cycle dependence	s(duty_*c*, *k*=10)	60.8	5703.8	1836.4	<0.001
9	IPI dependence	s(IPI_*c*, *k*=10)	58.3	6390.6	2523.1	<0.001
10	No temporal parameter dependence	1 (no temporal predictor)	52.0	6648.7	2781.3	<0.001

All candidate models used the same 6454 trials in which directional alignment could be calculated during both the pre-switch and post-switch periods. Models followed the general formula SFI∼[temporal predictor(s)]+trial_order_*c*+switch_direction+s(fly_id, bs=“re”). Trial order was the sequential stimulus presentation number within each fly's experiment. It was mean centred and divided by its standard deviation before model fitting and included as trial_order_*c*. The term switch_direction denotes LR versus RL speaker switching, and s(fly_id, bs=“re”) is a random-effect smooth for fly identity. Continuous temporal predictors denoted by the suffix _*c* were also mean centred and divided by their standard deviations. One-dimensional smooths are denoted by s(), whereas te() denotes a tensor product smooth used to estimate a two-dimensional response surface. The argument *k* specifies the basis dimension: one value for a one-dimensional smooth and one value for each margin of a tensor-product smooth. Inverse-frequency pulse duration–interpulse interval (PD–IPI) cell weights, rescaled to have a mean of one, were identical across candidate models. Models were fitted by maximum likelihood. The reported d.f. values are the model comparison degrees of freedom returned with the mgcv model log-likelihood. ΔAIC was calculated from unrounded AIC values relative to the lowest AIC model, and Akaike weights represent relative support within the full candidate set. The leading model was subsequently refitted using restricted maximum likelihood for inference and visualisation.

All candidates followed the general formula SFI∼[temporal predictor(s)]+trial_order_*c*+switch_direction+s(fly_id, bs=“re”), with the temporal predictor terms specified in Table 1. Pulse period was calculated as PD+IPI, and duty cycle was calculated as PD/(PD+IPI). Because pulse rate is the reciprocal of pulse period, separate pulse rate and pulse period models would provide redundant one-dimensional descriptions; pulse period was therefore used in the candidate set, whereas pulse rate was retained for descriptive comparison with previous studies.

Candidate models were fitted using maximum likelihood and ranked using Akaike's information criterion (AIC). We report model-comparison degrees of freedom, AIC, ΔAIC relative to the leading model and Akaike weights for the full candidate set. The leading temporal structure was subsequently refitted using restricted maximum likelihood for inference and visualisation (see below). Robustness to uneven sampling was assessed by repeating the complete candidate comparison after excluding the frequently repeated PD=10 ms, IPI=10 ms reference condition.

All behavioural data preprocessing and initial analyses were performed in MATLAB (R2019a, The MathWorks Inc.). Statistical analyses and model fitting were conducted in R (version 4.5.3).

To provide ecological context for understanding Floridian *O. ochracea* song recognition relative to their associated host species in Florida, we measured temporal parameters (PD and IPI) of calling songs for four documented Florida host species (Walker, 1993). For each species, PD and IPI were summarised as the mean±s.d. across five recordings. Species means were overlaid on the population-level prediction surface from the selected PD–IPI GAMM (Fig. S3).

## RESULTS

### SFI captures song preference behaviour

**Fig. 2.**
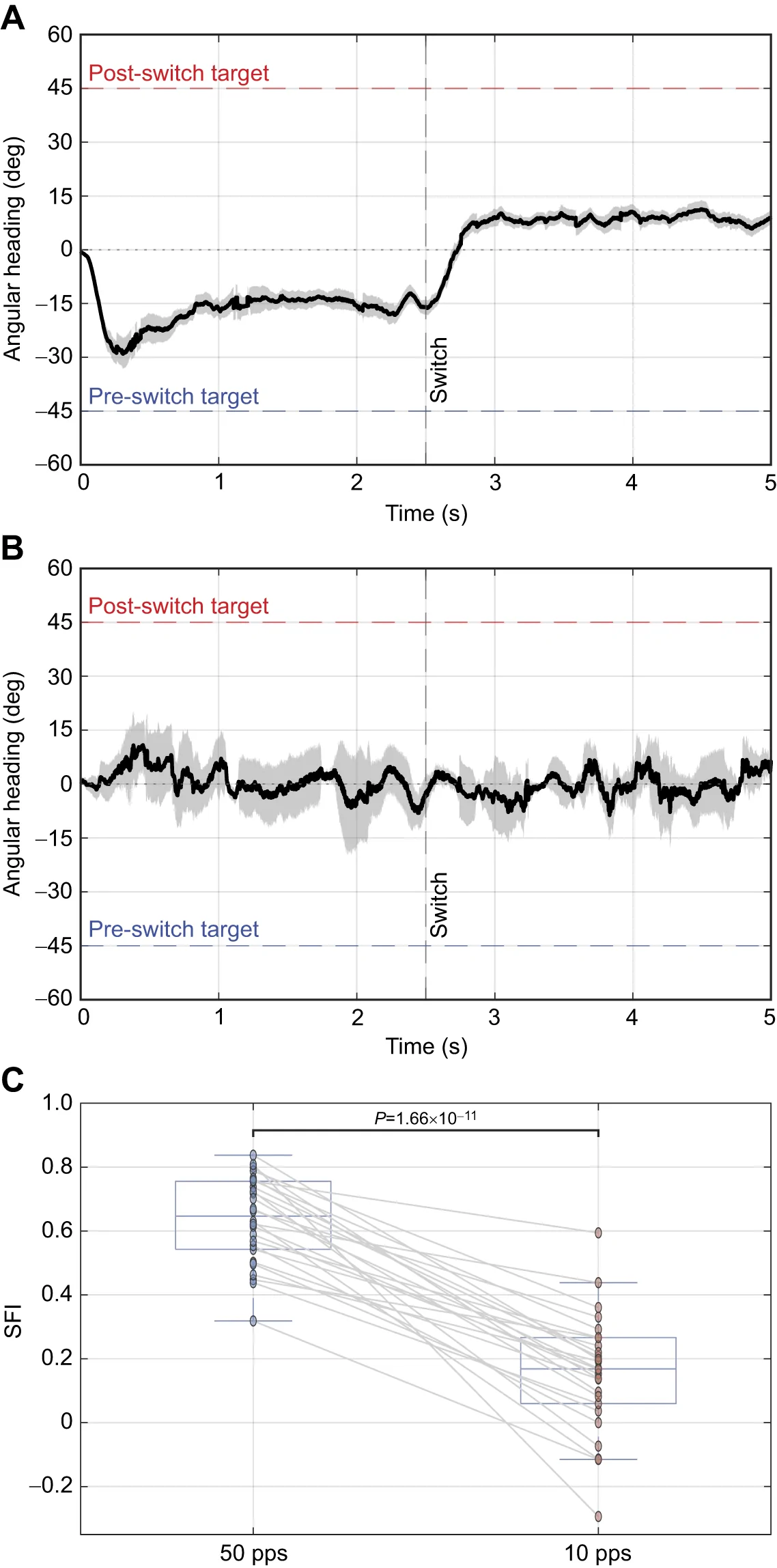
**Switch-following paradigm reveals song-specific tracking behaviour.** (A,B) Circular mean angular heading over time for all left-to-right (LR) trials using the 50 pulses s^−1^ preferred song (PD=10 ms, IPI=10 ms; 667 trials from 52 flies) and the 10 pulses s^−1^ song (PD=50 ms, IPI=50 ms; 23 trials from 22 flies), respectively. Trials were first averaged within flies at each time point in the response using a circular mean. The black line is the across-fly circular mean, and grey shading shows ±s.e.m. across fly-level means. The dashed horizontal lines indicate the pre-switch (−45 deg, blue) and post-switch (+45 deg, red) speaker directions, and the vertical dashed line marks the speaker switch at 2.5 s. (C) Switch-following index (SFI) for the same songs. SFI was calculated only for trials in which directional alignment could be estimated during both the pre-switch and post-switch periods after excluding heading measurements made while walking speed was below 0.2 cm s^−1^. Points connected by grey lines show the paired fly-level means for the 26 flies with qualifying trials for both songs; boxes show the median, upper and lower quartiles and 1.5× the interquartile range. SFI was greater for 50 pulses s^−1^ (mean±s.e.m. 0.635±0.027) than for 10 pulses s^−1^ (0.156±0.036; paired *t*-test: *t*_25_=11.54, *P*=1.66×10^−11^). pps, pulses per second.

To validate the SFI, we compared phonotactic responses to the preferred 50 pulses s^−1^ song and the non-preferred 10 pulses s^−1^ song. In response to the 50 pulses s^−1^ stimulus, flies consistently oriented towards the pre-switch speaker and shifted their heading towards the newly active speaker following the change in broadcast location (Fig. 2A). In contrast, responses to the 10 pulses s^−1^ stimulus were more variable and showed little consistent reorientation following the speaker switch (Fig. 2B). Among the 26 flies for which SFI could be calculated for both stimuli, mean SFI was greater for the 50 pulses s^−1^ stimulus (mean±s.e.m. 0.635±0.027) than for the 10 pulses s^−1^ stimulus (0.156±0.036; paired *t*-test: *t*_25_=11.54, *P*=1.66×10^−11^; Fig. 2C). Thus, SFI distinguished the directional tracking elicited by preferred and non-preferred song patterns among trials in which alignment could be calculated during both stimulus periods.

### Response availability varies across the stimulus space

Of the 9413 trials within the PD–IPI stimulus grid, directional alignment could be calculated during both the pre-switch and post-switch periods in 6454 trials (68.6%). Of the remaining trials, 1251 provided alignment only before the switch, 309 only after the switch, and 1399 during neither period. Accordingly, SFI analyses were restricted to trials with directional alignment values during both periods, whereas response availability was analysed separately using all trials.

Response availability varied strongly across the PD–IPI stimulus space (Fig. S1; tensor-product smooth: effective degrees of freedom, edf=44.11, χ^2^=1085, *P*<2×10^−16^). Availability also declined with trial order (log-odds estimate=−0.462 per standard deviation of trial order, s.e.=0.028, *z*=−16.39, *P*<2×10^−16^), whereas speaker-switch direction had no detectable effect (estimated RL–LR log-odds difference=0.0547, *P*=0.782). The availability model explained 23.1% of the deviance. Thus, failure to obtain directional alignment during both periods was stimulus dependent.

### Behavioural responses form a structured tuning surface in PD–IPI space

**Fig. 3.**
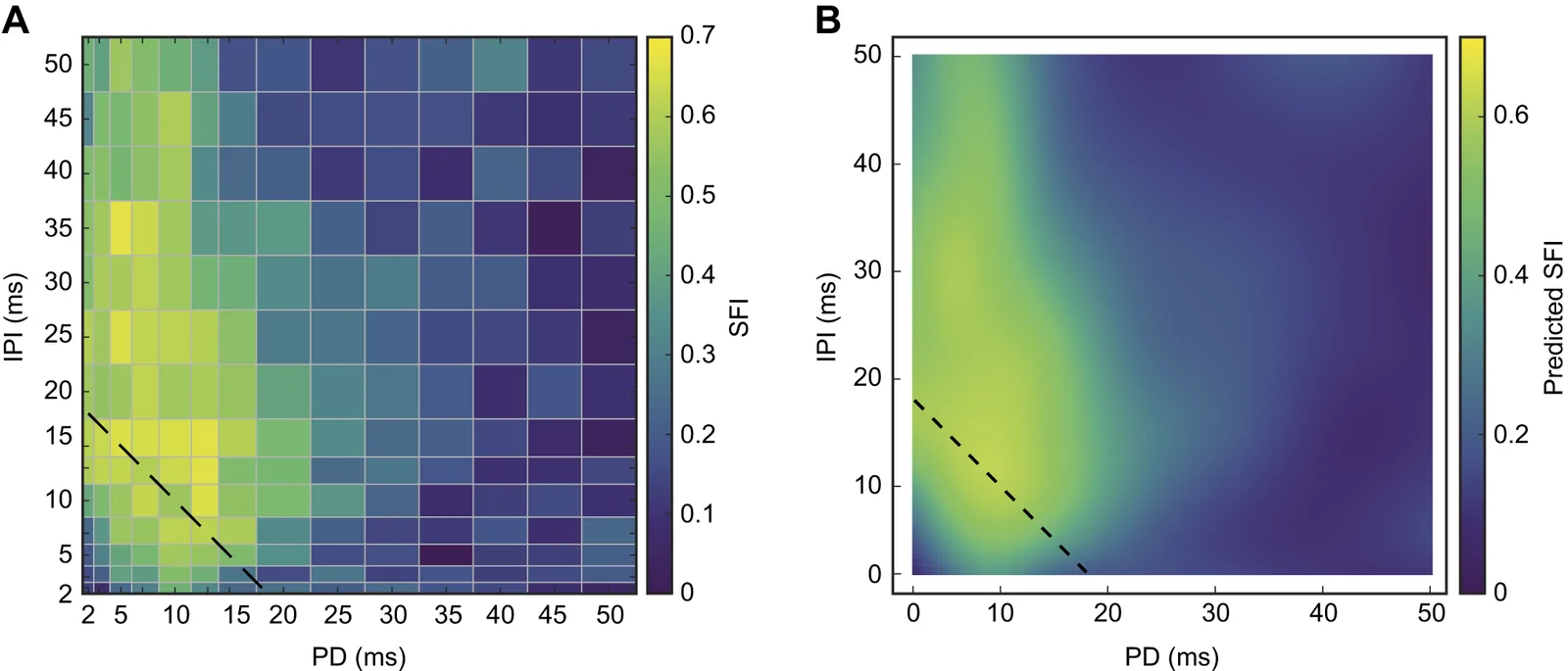
**Behavioural tuning reveals multidimensional temporal sensitivity.** (A) Mean SFI for each tested combination of PD and IPI. SFI was included only for trials in which directional alignment could be calculated during both the pre-switch and post-switch periods. Repeated trials were first averaged within fly and stimulus, after which means were calculated across flies (*n*=14–52 flies per stimulus combination). Cell dimensions reflect the numeric spacing between the tested PD and IPI values. (B) Population-level predictions from the leading joint PD–IPI generalised additive mixed model refitted using REML. Predictions exclude the fly identity random effect, set standardised trial order to zero (its mean) and use LR as the reference switch direction. In both panels, the dashed line marks PD+IPI=20 ms (50 pulses s^−1^), and the colour scale spans SFI=0–0.7.

To visualise how PD and IPI jointly shaped behavioural responses, we constructed an empirical heatmap of mean SFI across the 196 tested PD–IPI combinations (Fig. 3A). Repeated trials were first averaged within fly and stimulus, after which means were calculated across flies. The number of flies contributing to individual stimulus combinations ranged from 14 to 52.

Responses were structured across the stimulus space rather than distributed uniformly. Higher SFI values were concentrated primarily at PDs of approximately 5–15 ms, but the magnitude and distribution of these responses varied with IPI. Elevated responses were not confined to the dashed PD+IPI=20 ms line, indicating that stimulus combinations producing the same 50 pulses s^−1^ rate did not necessarily evoke equivalent responses. Thus, the empirical surface could not be described by a simple preference for one pulse rate, pulse period, PD or IPI. We tested these alternative temporal descriptions formally using the candidate GAMMs described below.

### Model comparison supports multidimensional dependence on temporal parameters

Model comparison strongly supported models describing joint two-dimensional response surfaces over one-dimensional and additive alternatives (Table 1). The joint PD–IPI response surface model had the lowest AIC (AIC=3867.4, Akaike weight=0.753). The joint duty cycle period response surface was the only competitive alternative (AIC=3869.7, ΔAIC=2.2, Akaike weight=0.247). All remaining candidate models had ΔAIC values of at least 242.6 and negligible Akaike weights. In particular, the PD-only model received substantially less support than either joint response surface model (ΔAIC=788.4), indicating that the pronounced dependence on PD did not, by itself, account for the full behavioural response surface.

The leading PD–IPI model was refitted using REML for inference and visualisation. Its predicted response surface reproduced the principal structure of the empirical response surface (Fig. 3B). The model explained 47.0% of the deviance (adjusted *R*^2^=0.458), and the joint PD–IPI smooth was strongly supported (edf=44.14; *F*=66.89; *P*<2×10^−16^).

SFI declined slightly with trial order (estimated change=−0.00886 per standard deviation of trial order, *P*=0.00855), although the magnitude of this effect was small. This corresponds to a predicted decline of approximately 0.0066 SFI units over 50 successive stimulus presentations. Speaker-switch direction had no detectable effect on SFI (estimated RL–LR difference=0.00627, *P*=0.792).

Although the PD–IPI response surface received approximately three-quarters of the Akaike weight, the support for the duty cycle period surface cautions against interpreting PD and IPI as the unique temporal coordinates represented by the nervous system. These models provide alternative coordinate descriptions of the same two-dimensional stimulus space. Thus, the model comparison indicates that behavioural recognition cannot be reduced to a single temporal descriptor, but it does not establish which temporal variables are explicitly encoded by the nervous system.

Excluding the frequently repeated PD=10 ms, IPI=10 ms reference condition did not change the model ranking. The PD–IPI response surface remained the leading model (Akaike weight=0.629), followed by the duty cycle period response surface (ΔAIC=1.05, Akaike weight=0.371). Thus, the leading model ranking was not driven by oversampling of the reference stimulus.

### PD and IPI effects are interdependent

**Fig. 4.**
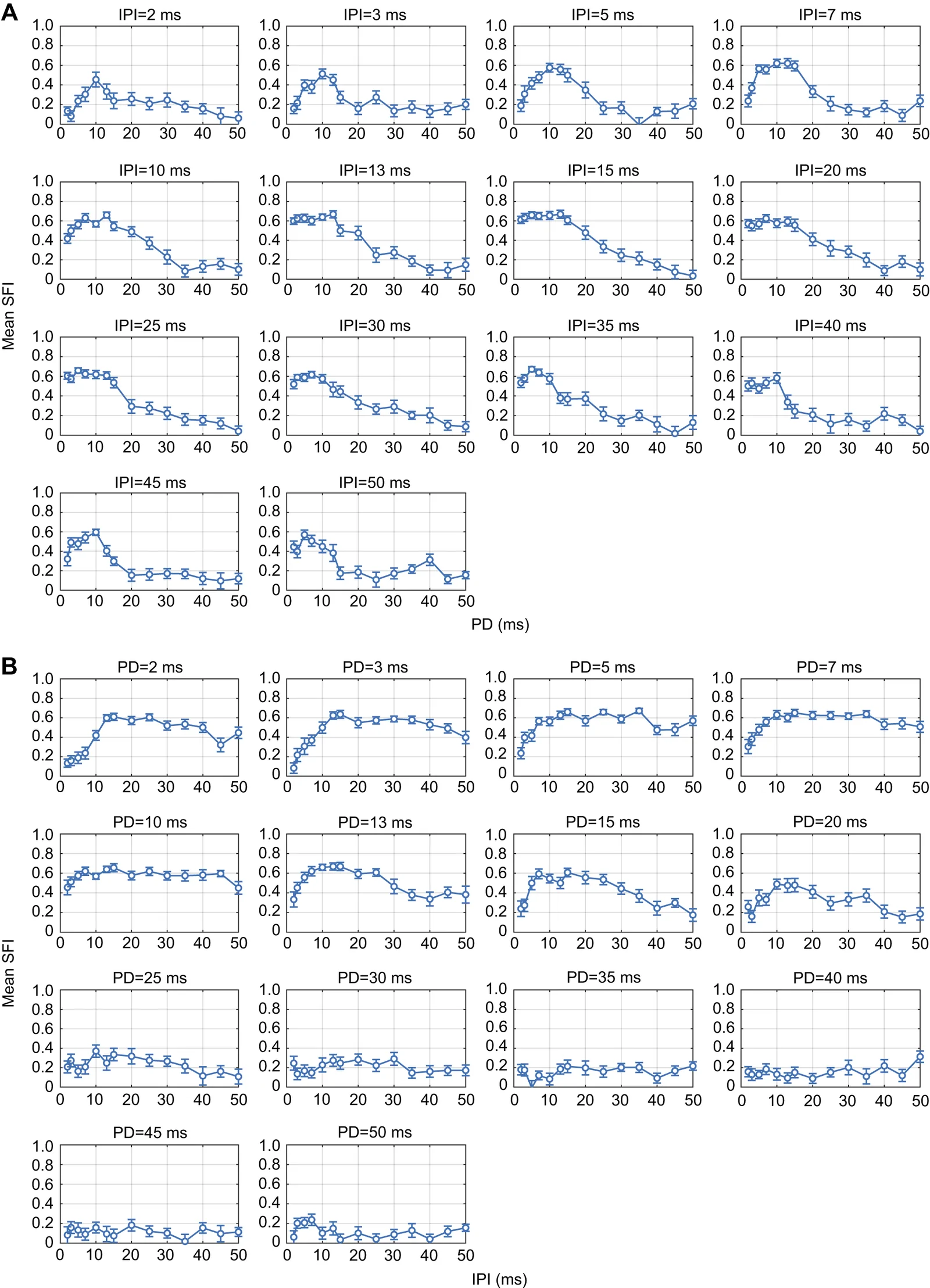
**PD and IPI tuning are interdependent.** (A) Mean SFI as a function of PD for each fixed IPI. (B) Mean SFI as a function of IPI for each fixed PD. SFI was included only for trials in which directional alignment could be calculated during both the pre-switch and post-switch periods. Repeated trials were first averaged within fly and stimulus, after which points and error bars were calculated as the mean±s.e.m. across flies (*n*=14–52 flies per stimulus combination). Lines connect tested stimulus combinations as visual guides. All panels use the same axis limits. These conditional views of the empirical response surface show that the apparent effect of either temporal parameter depends on the value of the other.

Consistent with the model results, we next examined behavioural responses along each temporal dimension while holding the other constant (Fig. 4). These conditional plots provide complementary views of the empirical PD–IPI response surface shown in Fig. 3A.

Across fixed IPIs, the highest SFI values generally occurred at PDs of approximately 5–15 ms (Fig. 4A). However, the magnitude, breadth and shape of PD tuning varied with IPI. Shorter PDs of 2–3 ms also elicited moderate-to-high responses at some intermediate and longer IPIs, whereas responses generally declined at PDs of 20 ms or longer. Thus, PD strongly structured behavioural responses, but its effect depended on IPI and could not independently account for the full response surface.

The relationship between IPI and SFI likewise depended on PD (Fig. 4B). At PDs of approximately 5–15 ms, elevated responses extended across a relatively broad range of IPIs, although the shapes of the conditional response profiles differed among PDs. At PDs of 2–3 ms, responses generally increased from short to intermediate IPIs, whereas responses at PDs of 20 ms or longer were generally lower and more variable. Consequently, there was no single IPI preference that consistently described responses across PDs. Together, these conditional views illustrate the interdependence of PD and IPI identified by the GAMM comparison.

### Responses do not collapse across iso-rate and iso-period stimulus combinations

**Fig. 5.**
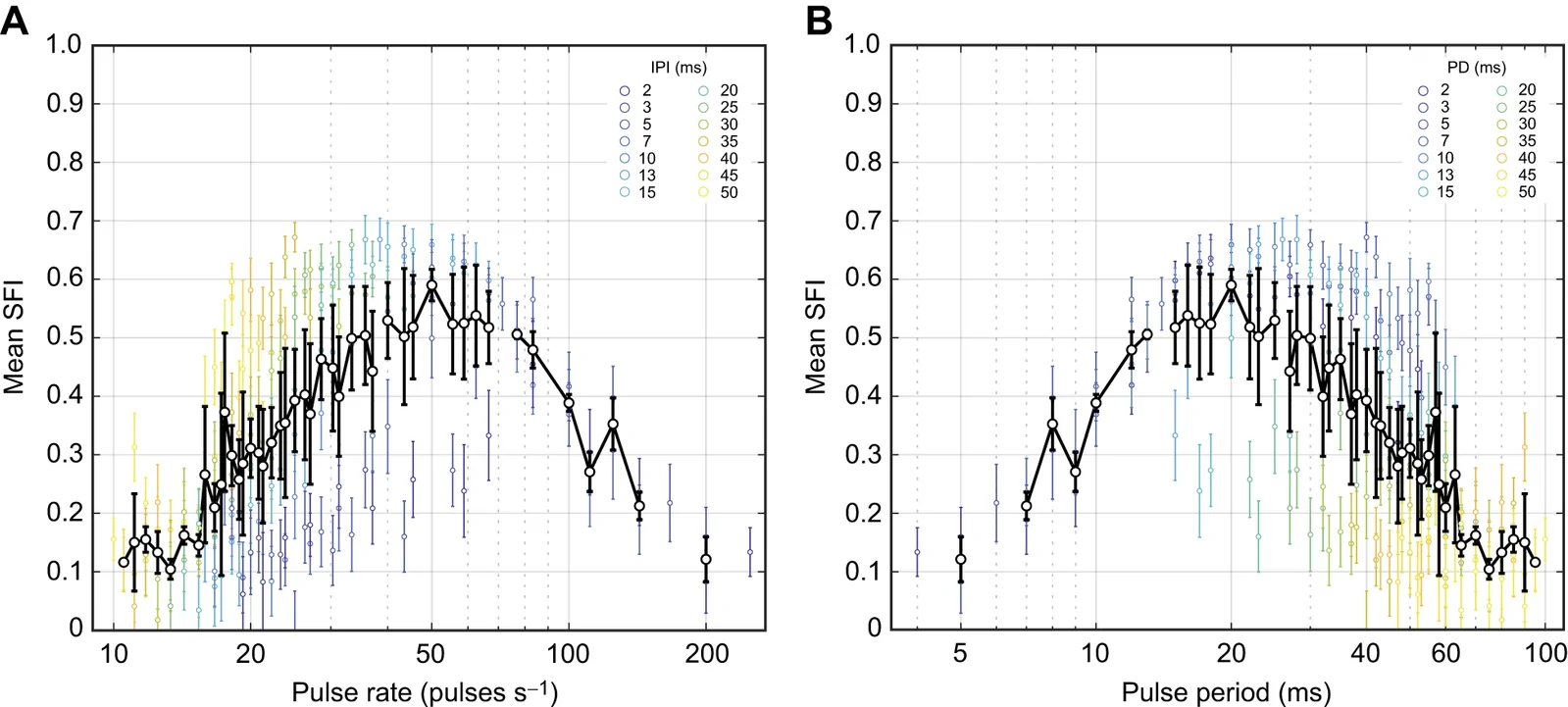
**Tests of iso-rate and iso-period collapse.** (A) Mean SFI for each tested PD–IPI stimulus combination plotted against pulse rate [1/(PD+IPI)]. Repeated trials were first averaged within fly and stimulus. Each coloured point and error bar represents the mean±s.e.m. across flies (*n*=14–52 per stimulus combination), with colour indicating the IPI. Black points show the mean across PD–IPI stimulus combinations that share the same pulse rate, black error bars show the s.e.m. among those combinations, and black lines connect these means. Black summaries are shown only when at least two distinct PD–IPI combinations produced the same rate. (B) The same stimulus combination means plotted against pulse period (PD+IPI), with colour identifying the PD. Pulse rate and pulse period are reciprocals, so A and B are alternative representations of the same stimulus combinations rather than independent analyses. The dispersion among stimulus combination means sharing a rate or period shows that responses do not collapse onto a single pulse rate or pulse period function.

To examine whether the behavioural response could be summarised by a single temporal descriptor, we plotted the empirical PD–IPI stimulus cell means as functions of pulse rate and pulse period (Fig. 5). Because pulse rate is the reciprocal of pulse period, Fig. 5A,B contains the same stimulus combinations expressed on reciprocal scales rather than two independent analyses. Pulse rate facilitates comparison with previous descriptions of a preference near 50 pulses s^−1^ in *O. ochracea* (Jirik et al., 2023; Lee et al., 2019; Walker, 1993), whereas pulse period directly represents the PD+IPI relationship used to construct the stimulus space.

Responses varied substantially among PD–IPI combinations sharing the same pulse rate or pulse period. For example, among combinations with a 40 ms pulse period (25 pulses s^−1^), mean SFI ranged from approximately 0 to 0.67. The mean response across stimulus combinations was highest near 50 pulses s^−1^ (20 ms period), but the individual stimulus combination means at that rate ranged from 0.499 to 0.659. Thus, an apparent peak in the one-dimensional average did not imply that all stimulus combinations producing that rate or period were behaviourally equivalent.

This lack of collapse is consistent with the candidate model comparison: neither pulse period nor, equivalently, pulse rate alone adequately described behavioural responses. Plotting responses against either scalar descriptor obscures variation associated with the particular combination of PD and IPI. Together with the joint response surface models, these empirical comparisons support multidimensional temporal dependence.

## DISCUSSION

Our results demonstrate that temporal pattern recognition in Floridian *O. ochracea* cannot be explained by a simple pulse rate detection mechanism. Consistent with prior work, the mean response across stimulus combinations was highest near 50 pulses s^−1^, corresponding to a pulse period of 20 ms (Jirik et al., 2023; Lee et al., 2019; Walker, 1993). However, responses did not collapse across PD–IPI combinations producing the same pulse rate or pulse period (Fig. 5). Stimuli with identical rates or periods were therefore not behaviourally equivalent, which is inconsistent with recognition models whose output depends solely on one of these scalar descriptors. In Floridian *O. ochracea*, song temporal pattern recognition cannot be reduced to pulse rate or pulse period alone.

Instead, multiple lines of evidence indicate that the behavioural response has a multidimensional temporal structure. First, the empirical and model-predicted response surfaces (Fig. 3), together with the conditional tuning analyses (Fig. 4), reveal structured tuning that cannot be captured by a single axis. A particularly prominent feature is the elevated response to PDs of approximately 5–15 ms across a broad range of IPIs, suggesting that PD places an important constraint on song recognition. However, responses within this PD range varied with IPI, indicating that PD alone was insufficient to account for the behavioural response. Second, responses did not collapse across stimuli sharing the same pulse period and, consequently, the same pulse rate (Fig. 5). Third, models estimating joint two-dimensional response surfaces substantially outperformed one-dimensional and additive models (Table 1). The joint PD–IPI response surface was the leading candidate, receiving an Akaike weight of 0.753, whereas the duty cycle period response surface remained a competitive alternative with a weight of 0.247. These models provide alternative coordinate descriptions of the same two-dimensional stimulus space. Thus, our results constrain the computation underlying recognition without establishing that PD and IPI are themselves independently encoded by the auditory system.

SFI quantified directional tracking only for trials in which alignment could be calculated during both the pre-switch and post-switch periods. Because response availability varied across the stimulus space (Fig. S1), SFI and response availability describe complementary aspects of behaviour: whether a trial provided usable movement measurements during both periods, and how accurately flies followed the speaker transition when it did. Conclusions based on SFI therefore apply specifically to trials that met this movement criterion.

Several neural computational approaches could potentially generate this multidimensional behavioural response. Relatively simple filters, including circuits based on sequential excitation and inhibition, can generate complex response surfaces when evaluated across PD–IPI stimulus space without requiring PD and IPI to be represented explicitly. Classical models of temporal processing, such as delay-line and coincidence-detection circuits (Jeffress, 1948), provide a useful starting point for understanding the neural mechanisms of song pattern recognition (Kostarakos and Hedwig, 2015; Schoneich et al., 2015). In terms of pulse rate recognition, an acoustic feature detector sensitive to pulse rates may arise from detectors that respond best when subsequent sound pulses arrive simultaneously at a coincidence detector. This can be achieved if auditory neurons physiologically introduce a temporal delay matching the salient IPI for a specific preferred pulse rate (Schoneich et al., 2015). However, a mechanism in which recognition depends solely on pulse rate recognition would predict strong invariance across stimuli sharing the same pulse rate, which is inconsistent with our data (Fig. 5). More generally, computational modelling of song recognition circuits in insects has demonstrated that tuning to specific temporal features, including PD, IPI or pulse rate, can emerge from modifications of circuit parameters even in the absence of an explicit coincidence-detection mechanism (Clemens et al., 2021). This suggests that similar tuning functions do not uniquely specify the underlying neural mechanism. Temporal pattern processing may occur across multiple stages (Hennig et al., 2014; Schildberger, 1984), with temporal selectivity emerging through the interplay of excitation and inhibition (Edwards et al., 2008; Leary et al., 2008) or temporally offset integration windows (Hedwig and Sarmiento-Ponce, 2017; Schoneich et al., 2015). Such mechanisms could generate the observed multidimensional behavioural response without requiring PD and IPI to be independently represented by the auditory system. Thus, the behavioural response surface constrains, but does not uniquely identify, the neural computations underlying temporal pattern recognition in *O. ochracea*.

While song pulse rate influences the phonotactic behaviour of Floridian *O. ochracea*, failure of iso-rate and iso-period collapse indicates that pulse rate, or its reciprocal pulse period, does not capture the full structure of behavioural recognition. The prominent responses to PDs of approximately 5–15 ms across a broad range of IPIs further indicate that PD places an important constraint on recognition. However, responses within this PD range vary with IPI, demonstrating that PD alone is also insufficient. Instead, we propose that the apparent pulse rate preference can be understood as a projection of the underlying multidimensional behavioural response surface. Specifically, different combinations of PD and IPI that fall within a behaviourally permissive region of this response space will necessarily correspond to a range of pulse rates and an apparent pulse rate preference when phonotactic responses are plotted along this single dimension (Jirik et al., 2023; Lee et al., 2019; Walker, 1993). In this sense, pulse rate can be a useful descriptive variable, but it does not capture the full structure of the behavioural response. This interpretation highlights the limitations of one-dimensional preference functions (Kilmer et al., 2017), which can obscure preferences based on multiple temporal dimensions, or any latent preferences not revealed by a single temporal descriptor (Gray et al., 2016).

Temporal pattern recognition has been studied in the intended receivers of a range of acoustically communicating species (Baker et al., 2019; Gerhardt and Huber, 2002). In many of these systems, signal recognition is described in terms of sensitivity to specific temporal parameters such as PD (Hennig, 2003), IPI and pulse rate (Doherty, 1985; Hennig, 2003; Thorson et al., 1982). For example, in two closely related cricket species, *Teleogryllus oceanicus* is selective for a range of pulse rates, while *Teleogryllus commodus* exhibits selectivity for PD (Hennig, 2003). Similarly, female Cope's grey tree frog, *Hyla chrysoscelis*, also exhibits selectivity for advertisement calls with pulse rates that match those produced by conspecific males (Lee et al., 2017; Schul and Bush, 2002). However, not all intended receivers can be described by tuning to a single temporal parameter. In some species, behavioural responses span a broad range of PD and IPI combinations, which suggests sensitivity to a combination of temporal parameters rather than a single derived metric. For instance, despite male *Hyla versicolor* producing advertisement calls with a specific pulse rate, females exhibit preferences for a broad range of PD and IPI that comprise a broad rectangular recognition space (Schul and Bush, 2002). Similarly, the response surface of Floridian *O. ochracea* includes substantial variation among stimuli sharing the same pulse rate, indicating that responses depend on combinations of temporal values rather than pulse rate alone. Thus, temporal pattern recognition can range from relatively low-dimensional parameter tuning to more complex multidimensional integration of temporal parameters.

Several limitations of the present study point to future directions. First, as our conclusions are based on behavioural data, direct tests of mechanisms of song recognition will depend on neurophysiological recordings combined with neuroanatomical results throughout the auditory pathway. If the multidimensional behavioural response originates within auditory processing, neural responses should differ among iso-rate or iso-period stimuli that evoke different behavioural responses. If such stimuli instead evoke similar auditory responses, the behavioural differences may emerge during subsequent sensorimotor transformations. Both female *O. ochracea* and female crickets depend on recognising and localising cricket calling songs to find their host or mate, respectively. Taking a comparative approach to characterise similarities or differences in their auditory processing can help shed light on common or diverse solutions to shared problems (Gerhardt and Huber, 2002).

Second, although all 196 stimulus combinations were represented, individual flies experienced subsets of the stimulus grid, and the PD=10 ms, IPI=10 ms reference condition was presented more frequently than other combinations (Fig. S2). Inverse-frequency weighting and a sensitivity analysis excluding this reference condition produced the same leading model ranking, reducing concern that the principal result was driven by uneven sampling. The broad stimulus grid was designed to distinguish among alternative recognition hypotheses and includes combinations likely to fall outside the variation found in natural host songs. The full response surface should therefore be interpreted as a diagnostic description of temporal recognition rather than as a representation of the natural distribution of host song parameters.

Third, *O. ochracea* occurs across parts of southern USA, the Hawaiian Islands and in Mexico (Gray et al., 2019; Lehmann, 2003). As *O. ochracea* from different regions have geographically variable host associations and respond to host songs with different temporal characteristics (Gray et al., 2007), it remains unknown whether song recognition is based on the same combination of temporal parameters described in the current study. To place the experimental stimulus space in an ecological context, we plotted the temporal characteristics of calling songs from four cricket species associated with *O. ochracea* in Florida over the model-predicted behavioural response surface (Fig. S3). These four species include: *G. rubens*, which is the dominant host throughout Florida; *Gryllus texensis*, which is the dominant host in Texas, but whose range extends into the western panhandle region of Florida; *Gryllus assimilis*, which has been introduced into Florida and is principally found in southern and central Florida; and *Gryllus firmus*, which is abundant and widespread in Florida. The songs of *G. texensis*, *G. assimilis* and *G. rubens* fall within regions of elevated predicted SFI, whereas *G. firmus* occupies a region associated with lower predicted responses. Notably, *G. firmus* accounted for only ∼3% of *O. ochracea* captured in sound traps in Florida (Walker, 1993), suggesting that its position within a lower-response region may be consistent with its comparatively limited association with flies in the field. More broadly, the correspondence between the temporal characteristics of commonly associated host songs and regions of elevated behavioural response raises the possibility that the recognition landscape accommodates temporal variation among multiple hosts, a hypothesis that will require direct comparative testing with natural songs.

Fourth, in Hawaii, where both *O. ochracea* and a novel cricket host, *Teleogryllus oceanicus*, have been introduced, the novel host cricket calling songs of *T. oceanicus* have been found to be rapidly evolving in response to selection from flies (Gallagher et al., 2023; Tinghitella et al., 2018, 2021). These novel songs are more cryptic and differ in frequency content and fine-scale temporal features compared with the ancestral calling songs (Tinghitella et al., 2018). Likely in response to host availability or the cryptic nature of novel songs, some populations of Hawaiian *O. ochracea* have also been found to switch hosts and to rely on multiple host species (Broder et al., 2023). These changes suggest that some level of flexibility in recognising temporal features would be adaptive for Hawaiian *O. ochracea*.

In summary, our results demonstrate that temporal pattern recognition in Floridian *O. ochracea* cannot be explained by a simple pulse rate detection mechanism. Instead, phonotactic responses form a multidimensional temporal response surface that cannot be reduced to pulse rate (or pulse period) or another single scalar descriptor. PD places a strong constraint on behavioural responses, but its effects vary with IPI, indicating that neither PD nor pulse rate alone fully describes song recognition. Although independently varying PD and IPI revealed this structure, these stimulus dimensions should not necessarily be interpreted as features explicitly represented by the auditory system. Multiple neural computations, including relatively simple temporal filters, could generate the observed behavioural surface. Within this framework, apparent pulse rate tuning arises as a one-dimensional projection of a more complex response surface rather than demonstrating that pulse rate is the sole recognition variable. Similar principles may underlie pattern recognition across a range of acoustic communication systems, including both intended receivers and eavesdroppers.

## Supplementary Material

10.1242/jexbio.252806_sup1 Supplementary information
