# Supplementary material for: Song recognition in Floridian Ormia ochracea reflects multidimensional temporal tuning
Source: J Exp Biol. 2026 Sep 15;229(18):jeb252806. doi: 10.1242/jeb.252806 (PMC13615676; doi:10.1242/jeb.252806)
Supplement: Supplementary file 1 [file jexbio-229-252806-s1.pdf]

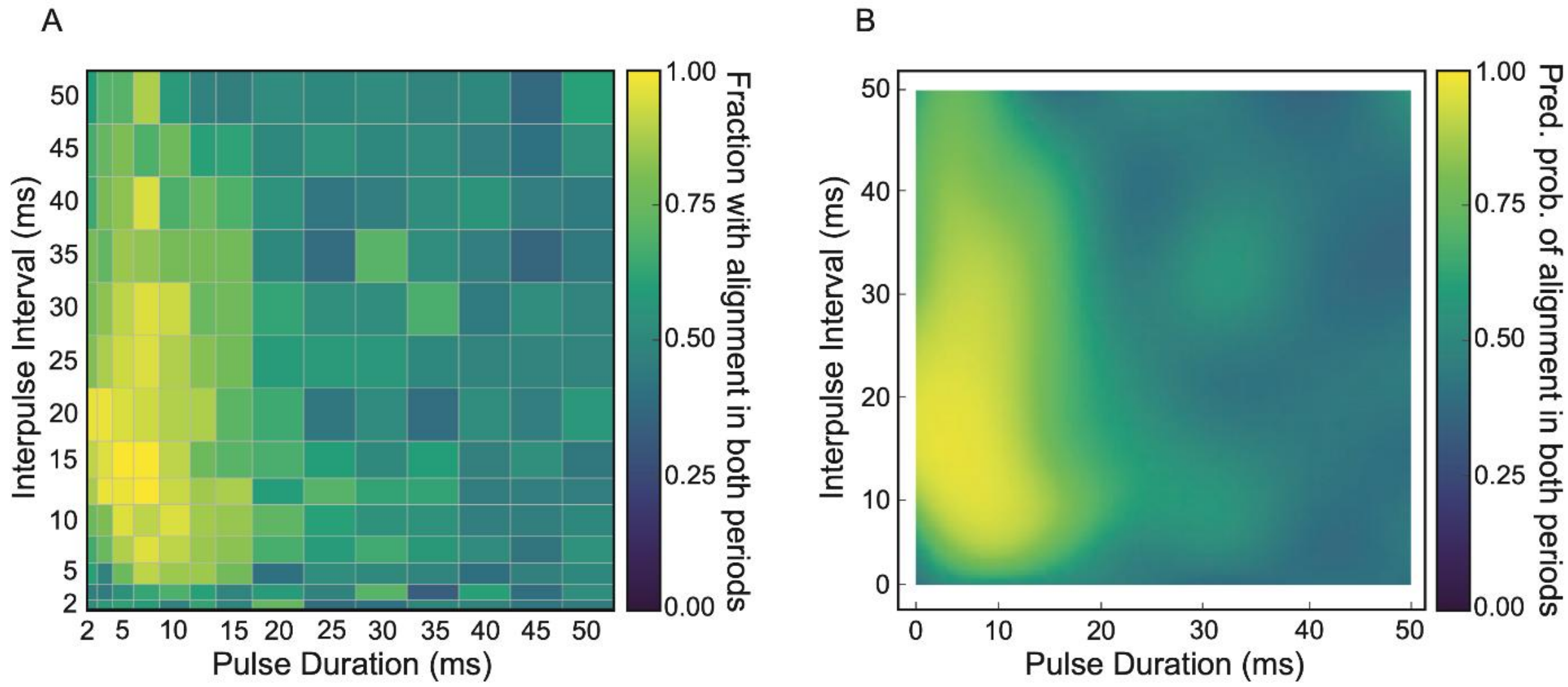

Fig. S1. Response availability varies across the PD–IPI stimulus space. (A) Observed fraction of trials in which directional alignment could be calculated during both the pre-switch and post-switch periods for each PD–IPI stimulus combination. (B) Population-level predictions from a binomial PD–IPI generalised additive mixed model fitted to all 9,413 trials. Predictions exclude the fly-identity random effect, set standardised trial order to zero (its mean), and use LR as the reference switch direction. Both panels use a probability scale from zero to one. The probability that a trial provided alignment values for both periods varied across the stimulus space.

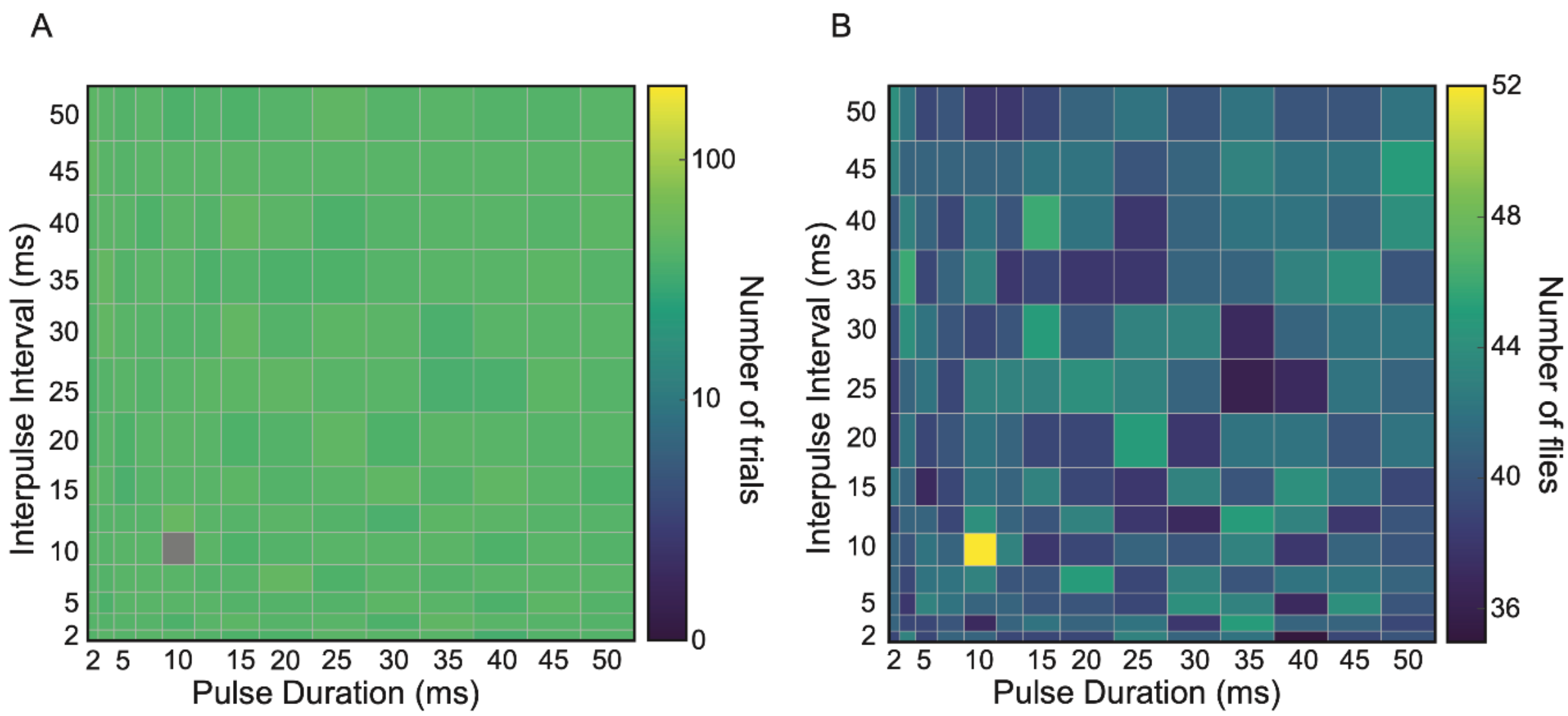

Fig. S2. Sampling density across the pulse duration–interpulse interval stimulus space. (A) Number of trials presented at each of the 196 pulse duration (PD) and interpulse interval (IPI) combinations on a logarithmic scale. To help visualize the sampling density of all PD-IPI combinations, the PD=10 ms, IPI=10 ms reference stimulus was de-emphasized from this sampling density as it was presented substantially more than the other combinations (1,245 trials for PD=10 ms, IPI=10 ms). The reference stimulus number of trials was de-emphasized to help better display the other PD-IPI combinations that were sampled less (approximately 40-50 trials for every PD=IPI combination). (B) Number of individual flies tested at each PD–IPI combination. The reference stimulus was represented by 1,245 trials from 52 flies; the remaining combinations were represented by 35–51 trials from 35–46 flies.

Journal of Experimental Biology • Supplementary information

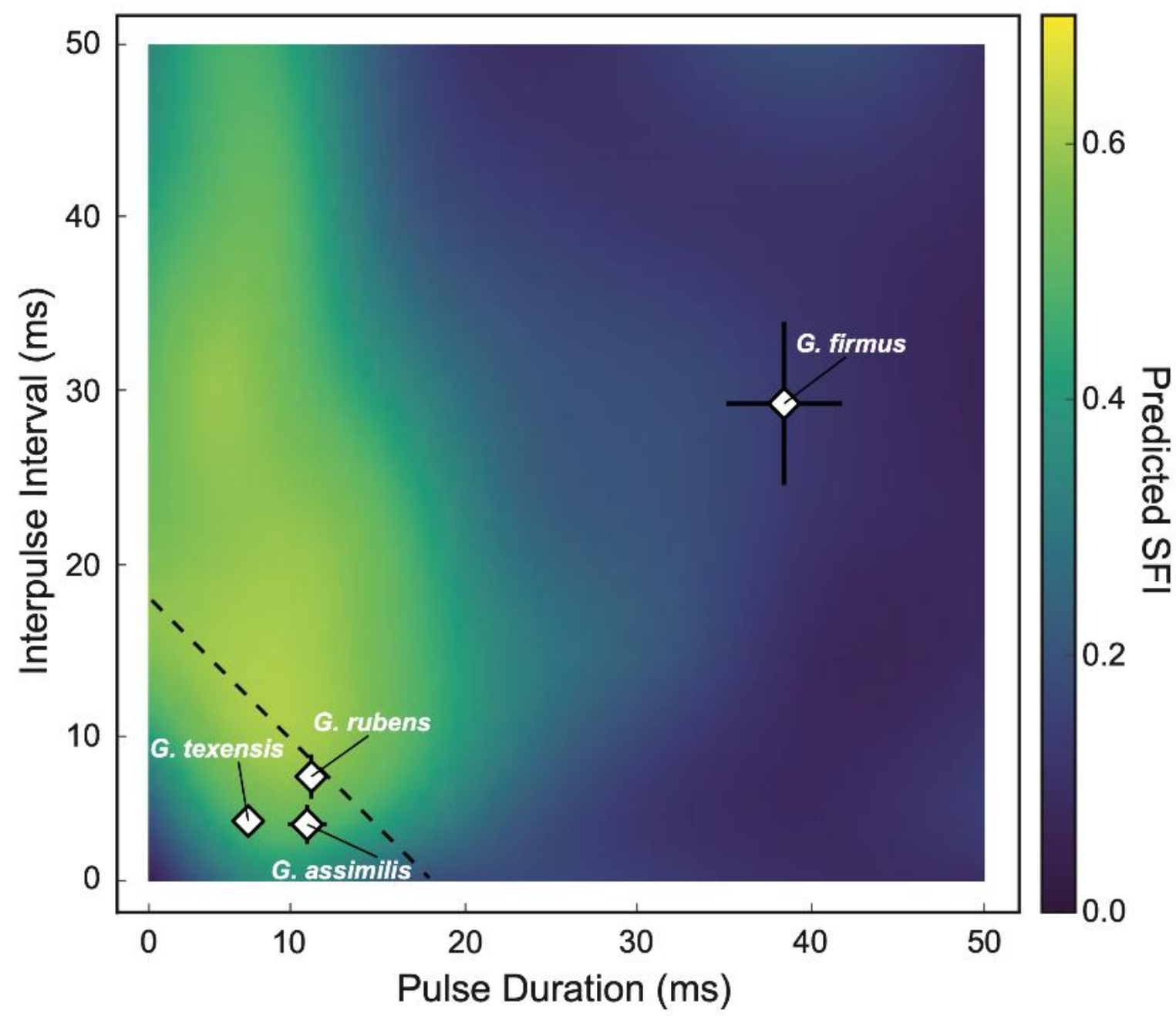

Fig. S3. Temporal characteristics of calling songs from documented Florida host species in relation to the behavioural response surface of Florida *Ormia ochracea*. White diamonds show the mean pulse duration (PD) and interpulse interval (IPI) of calling songs from four confirmed host Gryllus species of *Ormia ochracea* in Florida (Walker, 1993); horizontal and vertical bars show ±1 SD among five recordings per species. Background colour shows the switch-following index (SFI) predicted by the selected PD–IPI GAMM, using mean trial order and the LR reference switch direction and excluding the fly-specific random effect, as in Fig. 3B. The dashed diagonal denotes PD+IPI=20 ms, corresponding to 50 pulses s^{-1}. Host-song measurements are included in the accompanying data archive.
